# Supplementary material for: Safety and Efficacy of Tyrosine Kinase Inhibitors in Immune Thrombocytopenic Purpura: A Systematic Review of Clinical Trials
Source: J Xenobiot. 2023 Jan 28;13(1):29–41. doi: 10.3390/jox13010005 (PMC9944448; doi:10.3390/jox13010005)
Supplement: Supplementary file 1 [file jox-13-00005-s001.zip › jox-2164492-supplementary.pdf]

Table S1. Search strategy in this article.

P	I	C	O	S
"Purpura, Thrombocytopenic, Idiopathic"[Mesh]	"Protein-Tyrosine Kinases"[Mesh]			
Idiopathic Thrombocytopenic Purpura<br>Idiopathic Thrombocytopenic Purpuras<br>Immune Thrombocytopenic Purpura<br>Immune Thrombocytopenic Purpuras<br>Immune Thrombocytopenia<br>Immune Thrombocytopenias<br>Thrombocytopenic Purpura, Autoimmune<br>Werlhof Disease<br>Werlhof's Disease<br>Werlhofs Disease<br>Autoimmune Thrombocytopenia<br>Autoimmune Thrombocytopenias<br>Autoimmune Thrombocytopenic Purpura<br>Autoimmune Thrombocytopenic Purpuras	Protein Tyrosine Kinases<br>Tyrosine-Specific Protein Kinases<br>Tyrosine Specific Protein Kinases<br>Tyrosine Protein Kinase<br>Tyrosine Kinase<br>Tyrosine-Specific Protein Kinase<br>Kinase, Tyrosine-Specific Protein<br>Tyrosine Specific Protein Kinase<br>Tyrosylprotein Kinase<br>Tyrosine Protein Kinases<br>Protein-Tyrosine Kinase<br>Protein Tyrosine Kinase<br>Rilzabrutinib<br>Fostamatinib			
Pubmed:<br>(((((((((((((("Protein-Tyrosine Kinases"[Mesh]) OR (Protein Tyrosine Kinases)) OR (Tyrosine-Specific Protein Kinases)) OR (Tyrosine Specific Protein Kinases)) OR (Tyrosine Protein Kinase)) OR (Tyrosine Kinase)) OR (Tyrosine-Specific Protein Kinase)) OR (Kinase, Tyrosine-Specific Protein)) OR (Tyrosine Specific Protein Kinase)) OR (Tyrosylprotein Kinase)) OR (Tyrosine Protein Kinases)) OR (Protein-				

Tyrosine Kinase)) OR (Protein Tyrosine Kinase)) OR (Rilzabrutinib)) OR (Fostamatinib)) AND ((((((((((((("Purpura, Thrombocytopenic, Idiopathic"[Mesh]) OR (Idiopathic Thrombocytopenic Purpura)) OR (Idiopathic Thrombocytopenic Purpuras)) OR (Immune Thrombocytopenic Purpura)) OR (Immune Thrombocytopenic Purpuras)) OR (Immune Thrombocytopenia)) OR (Immune Thrombocytopenias)) OR (Thrombocytopenic Purpura, Autoimmune)) OR (Werlhof Disease)) OR (Werlhof's Disease)) OR (Werlhofs Disease)) OR (Autoimmune Thrombocytopenia)) OR (Autoimmune Thrombocytopenias)) OR (Autoimmune Thrombocytopenic Purpura)) OR (Autoimmune Thrombocytopenic Purpuras)) ------ 162
WOS: With keywords mentioned above. ---- 130
Ovid Embase: with keywords mentioned above. ---- 179
Clinicaltrials.gov: Tyrosine kinase + Immune thrombocytopenia
